# Adverse Effects of Polycystic Ovarian Syndrome on Pregnancy Outcomes in Women With Frozen-Thawed Embryo Transfer: Propensity Score-Matched Study

**DOI:** 10.3389/fendo.2022.878853

**Published:** 2022-06-06

**Authors:** Zhexin Ni, Shanshan Mei, Siting You, Yi Lin, Wen Cheng, Ling Zhou, Yanping Kuang, Chaoqin Yu

**Affiliations:** ^1^ Department of Traditional Chinese Medicine Gynecology, Changhai Hospital, Shanghai, China; ^2^ Department of Traditional Chinese Medicine Gynecology, Shanghai University of Traditional Chinese Medicine, Shanghai, China; ^3^ Central Laboratory, Changhai Hospital, Second Military Medical University, Shanghai, China; ^4^ Department of Traditional Chinese Medicine, Naval Medical University, Shanghai, China; ^5^ Department of Assisted Reproductive Medicine, Shanghai Ninth People’s Hospital, School of Medicine, Shanghai Jiao Tong University, Shanghai, China

**Keywords:** PCOS, FET, propensity score-matched study, pregnancy, hormone

## Abstract

**Purpose:**

This work aimed to evaluate the adverse effect of polycystic ovary syndrome (PCOS) on pregnancy outcomes of singletons after vitrification in women with frozen-thawed embryo transfer (FET).

**Methods:**

Patients with/without PCOS who underwent FET from January 2013 and December 2018 were included. Propensity score matching (PSM) was used to reduce the influence of bias. Logistic regression was applied to identify the risk factors of adverse pregnancy outcomes of singletons in women with PCOS.

**Result:**

After PSM, the PCOS group had shorter gestational age (P<0.001) and lower newborn birth weight than the non-PCOS group (P=0.045). Compared with the non-PCOS group, the PCOS group had an increased risk of gestational diabetes mellitus (GDM) and pregnancy-induced hypertension (PIH) (P<0.001), placenta and membrane abnormality (P<0.001), stillbirth (P<0.001), neonatal complication (P=0.014), and miscarriage rate (P<0.001). Neonatal complication was associated with parity (adjusted OR=1.202, 95% CI=1.002–1.443, P=0.048) and basal P level (adjusted OR=1.211, 95% CI=1.021–1.436, P=0.028). According to multivariable logistic regression analysis, the miscarriage rate was related to parity (adjusted OR=1.201, 95% CI=1.057–1.166, P=0.005) and basal E2 (adjusted OR=1.002, 95% CI=1.000–1.004, P=0.019) and P levels on the day of embryo transfer (adjusted OR=0.971, 95% CI=0.957–0.985, P<0.001).

**Conclusions:**

Compared with non-PCOS women, women with PCOS have a higher risk of GDM and PIH, and neonatal complications and therefore require additional care during pregnancy and parturition.

## Introduction

PCOS is the most common hormonal disorder in women of reproductive age and accounts for 80% of women with anovulatory infertility (1, 2). Upon exclusion of other specific diagnoses, PCOS is characterized by a combination of androgen excess and ovarian dysfunction. In women with PCOS, ineffectual aromatization to estrogens and increased androgen level lead to a low FSH level, resulting in androgen excess and estrogen shortage (3). Abnormal hormone levels in women with PCOS may lead to poor pregnancy outcomes (4, 5). As one of the clinical manifestations of PCOS, obesity is also related to poor pregnancy outcomes in women with PCOS (6) and is usually associated with high circulating insulin levels, which in turn increase ovarian androgen production (7). The abnormal hormones prevent women of reproductive age from ovulation, the main cause of infertility caused by PCOS.

*In vitro* fertilization (IVF) has been widely used in infertility treatment for decades. Compared with spontaneous pregnancies, IVF pregnancies in women with PCOS are associated with increased risks of adverse pregnancy outcomes (8, 9). During the treatment, the serum levels of hormones change dramatically. A previous research showed that frozen-thawed embryo transfer (FET) is associated with preeclampsia in infertile women with PCOS (10), and other studies found that sex hormones such as testosterone and FSH are associated with pregnancy outcomes in IVF treatment (11, 12). However, the adverse effect of PCOS on pregnancy outcomes in women with FET has never been clarified.

Therefore, this study aims to evaluate the adverse effect of PCOS on pregnancy outcomes in frozen embryo transfer cycles. The risk predictors of adverse pregnancy outcomes in women with PCOS were also identified. Propensity score matching (PSM) was performed to exclude the confounding bias between women with and without PCOS. A logistic regression model was established for the precise evaluation of the risks factors of adverse pregnancy outcomes in women with PCOS. The findings would serve as a basis for the implementation of management measures during routine clinical practice.

## Methods and Materials

### Study Population and Characteristics

Patients who underwent IVF/intracytoplasmic sperm injection (ICSI) with FET were identified, and the women who conceived singleton were selected. PCOS was diagnosed by two gynecologists on the basis of the 2003 Rotterdam criteria for the patients who met two of the following criteria (1): oligo- or anovulation, (2) clinical and/or biochemical signs of hyperandrogenism, and (3) polycystic ovaries and exclusion of other related etiologies (13). Patients with the following diseases were excluded: (1) congenital uterine malformations; (2) severe cerebrovascular, liver, heart, or kidney diseases; (3) gynecological cancers; (4) metabolic or endocrine disorders (diabetes or pituitary adenomas); and (5) autoimmune diseases, such as systemic lupus erythematosus or scleroderma. We only included the patients with fallopian tubal blockage or infertility couples due to paternal factors in the non-PCOS group to minimize the influence of confounding factors. Cases with missing information of cycles, embryo, and clinical pregnancy data were also excluded. The following clinical data were collected: maternal age, maternal BMI, paternal age, duration of infertility, parity, cycle method, sperm origin, fertilization method, scoring for cleavage-stage and blastocyst-stage embryo, number of embryo transferred, basal LH, basal E2, basal FSH, basal P, basal testosterone (T), serum levels of E2 and P on the day of embryo transfer, neonatal gender, gestational age, birth weight, ectopic pregnancy, GDM and PIH, placenta and membrane abnormality, birth defect, stillbirth, complications of labor and delivery, neonatal complication, and miscarriage. This study was approved by the Research Ethics Committee of the Ninth People’s Hospital, Shanghai Jiao Tong University School of Medicine, and Changhai Hospital, Naval Medical University and complied with the Declaration of Helsinki. Owing to the retrospective design of this study, informed consent was waived.

### Laboratory Protocols and Embryo Assessment

Blood samples for basal LH, E2, FSH, P, and T assessment were collected in the morning after overnight fasting, preferably on days 2–5 of the menstrual cycle of women with regular menstruation or during withdrawal bleeding in women with amenorrhea. Hormonal assays were performed with UniCel DxI 800 Access Immunoassay System (Beckman Coulter, Brea, CA) using commercial kits following the manufacturers’ protocol. E2 and P on the day of FET were measured to assess their effect on pregnancy outcomes. Conventional IVF or ICSI was conducted depending on semen parameters and previous fertilization histories. All embryos were incubated in oil under 5% O_2_, 6% CO_2_, and 37°C. Vitrification and thawing were performed as previously described (14). Embryo quality was assessed during cleavage (day 3) or blastocyst stage (day 5/6). The scoring system for cleavage-stage embryos was based on the Istanbul consensus workshop (15). The blastocysts was grouped into four categories based on inner cell mass and trophectoderm scoring (15–17).

### Statistical Analysis

All analyses were conducted in SPSS (version 26.0 IBM Corporation, Armonk, NY) and R software (http://www.r-project.org/). Student’s t-test was used to compare continuous variables, which were expressed as mean ± standard deviation (SD). Chi-squared test or Fisher’s exact test was employed to analyze categorical data. Kruskal–Wallis test was applied to assess the relationship between PCOS and embryo quality. Propensity score was used to match the following independent variables to balance the influence of confounding factors: maternal age, paternal age, sperm origin method, stage at cryopreservation, and number of embryos transferred. A 1:1 match between the PCOS group and the non-PCOS group was obtained by nearest neighbor matching with a caliper width of 0.01 and without replacement. PSM was performed with R software using the MatchIt package. Univariable and multivariable logistic regression analyses were carried out to identify risk factors such as maternal BMI, parity, cycle method, fertilization method, basal LH, basal E2, basal FSH, basal P, total T, and E2 and P on the day of embryo transfer in adverse pregnancy outcomes. Hazard ratio (HR) and 95% confidence interval (95% CI) were calculated to assess the relationship between serum levels and pregnancy outcomes. All P values were two-tailed, and <0.05 was considered statistically significant.

## Results

A retrospective cohort including 1384 patients with PCOS and 14606 patients without PCOS was enrolled at the Ninth People’s Hospital, Shanghai Jiao Tong University School of Medicine between January 2013 and December 2018. After PSM, 1376 patients with PCOS and 1376 patients without PCOS were included in the PCOS and non-PCOS groups, respectively. In the non-PCOS group, 1337 patients with fallopian tubal blockage and 39 patients with paternal factors. The baseline characteristics, PCOS-associated characteristics, and pregnancy outcomes of singletons conceived after FET before and after PSM were evaluated.

### Patient Baseline Characteristics

Before PSM, the baseline characteristics of the two groups were unevenly distributed. The maternal and paternal ages of the PCOS group were younger than those in the non-PCOS group (maternal age: 30.54 ± 3.51 *vs.* 32.61 ± 4.45, P<0.001; paternal age: 32.42 ± 4.32 *vs.* 34.53 ± 5.53, P<0.001). The spouses of the non-PCOS group were more inclined to testicular sperm extraction than ejaculation (P<0.001). After matching, the baseline characteristics of the two groups were not different except for the scoring of blastocyst-stage embryo. The maternal age, paternal age, parity, cycle method, sperm origin method, fertilization method, stage at cryopreservation, and number of embryos transferred were similar in the two groups as shown in **Table 1**.

**Table 1:** The baseline characteristics of singletons conceived after frozen/thawed embryo transfer (FET).

Characteristic	Before PSM (N=15990)			After PSM (N=2752)		
	PCOS group (N=1384)	Non-PCOS group (N=14606)	P	PCOS group (N=1376)	Non-PCOS group (N=1376)	P
Maternal Age (y, mean ± SD)	30.54 ± 3.51	32.61 ± 4.45	<0.001	30.58 ± 3.49	30.63 ± 3.60	0.711
Paternal age (y, mean ± SD)	32.42 ± 4.32	34.53 ± 5.53	<0.001	32.45 ± 4.31	32.35 ± 4.21	0.543
Sperm origin			<0.001			0.052
Ejaculation	1374 (99.3%)	14290 (97.8%)		1373 (99.8%)	1366 (99.3%)	
Testicular sperm extraction	10 (0.7%)	316 (2.2%)		3 (0.2%)	10 (0.7%)	
Stage at cryopreservation			0.991			0.152
Cleavage stage	1138 (82.2%)	12008 (82.2%)		1133 (82.3%)	1161 (84.4%)	
Blastocyst	246 (17.8%)	2598 (17.8%)		243 (17.7%)	215 (15.6%)	
Scoring for cleavage-stage embryo^*^			0.447			0.134
Good	151 (13.3%)	1776 (14.8%)		151 (13.3%)	136 (11.7%)	
Fair	830 (72.9%)	8240 (68.6%)		825 (72.8%)	845 (72.8%)	
Poor	157 (13.8%)	1992 (16.6%)		157 (13.9%)	180 (15.5%)	
Scoring for blastocyst-stage embryo^#^			0.097			0.023
Excellent	18 (7.3%)	178 (6.9%)		18 (7.4%)	10 (4.7%)	
Good	39 (15.9%)	377 (14.5%)		39 (16.1%)	26 (12.1%)	
Average	138 (56.1%)	1353 (52.1%)		136 (55.9%)	119 (55.3%)	
poor	51 (20.7%)	690 (26.6%)		50 (20.6%)	60 (27.9%)	
No. of embryos transferred			0.056			0.188
1	250 (18.1%)	2953 (20.2%)		248 (18.0%)	222 (16.1%)	
≥2	1134 (81.9%)	11653 (79.8%)		1128 (78%)	1154 (83.9%)	

*Scoring system for cleavage-stage embryos was based on the Istanbul consensus workshop; ^#^Blastocyst was grouped into four categories based on inner cell mass and trophectoderm scoring (15–17).

### PCOS-Associated Characteristics

After matching, the maternal BMI differed between the PCOS and non-PCOS groups (23.58 ± 6.08 *vs.* 21.53 ± 5.03, P<0.001). Non-PCOS patients used natural cycles, and those with PCOS employed artificial cycles (P<0.001). The parity of the non-PCOS group was higher than that of the PCOS group (P=0.038). The number of patients in the non-PCOS group who underwent IVF+ICSI or ICSI was significantly higher than that in the PCOS group (P<0.001).

For sex hormonal panels, the serum levels of basal LH, basal FSH, and total T were higher in the PCOS group than in the non-PCOS group (basal LH: 5.42 ± 3.92 *vs.* 4.62 ± 3.42, P<0.001; total T: 0.04 ± 0.13 *vs.* 0.03 ± 0.12, P=0.048). The serum concentration of basal E2 and basal FSH were lower in the PCOS group than in the non-PCOS group (basal E2: 39.51 ± 51.17 *vs.* 60.08 ± 62.69, P<0.001; basal FSH: 5.07 ± 1.41 *vs.* 6.02 ± 3.78, P<0.001). Other hormonal indicators such as basal P and serum E2 and P levels on the day of embryo transfer were not different between the two groups. All the PCOS-associated characteristics are summarized in **Table 2**.

**Table 2:** The PCOS-associated characteristics of singletons conceived after frozen/thawed embryo transfer (FET).

Characteristics	Before PSM (N=15990)			After PSM (N=2752)		
	PCOS group (N=1384)	Non-PCOS group (N=14606)	P	PCOS group (N=1376)	Non-PCOS group (N=1376)	P
Maternal BMI (Kg/m^2^, mean ± SD)	23.44 ± 3.74	21.65 ± 4.24	<0.001	23.58 ± 6.08	21.53 ± 5.03	<0.001
Duration of infertility (y, mean ± SD)	3.46 ± 2.55	3.41 ± 2.98	0.553	3.44 ± 2.49	3.37 ± 2.48	0.490
Parity			<0.001			0.038
first	917 (66.3%)	7618 (52.2%)		912 (66.3%)	860 (62.5%)	
High order	467 (33.7%)	6988 (47.8%)		464 (33.7%)	516 (37.5%)	
Cycle method			<0.001			<0.001
Natural cycle	45 (3.3%)	4021 (27.5%)		45 (3.3%)	381 (27.7%)	
Artificial cycle	1339 (96.7%)	10585 (72.5%)		1331 (96.7%)	995 (72.3%)	
Fertilization method			<0.001			<0.001
IVF	817 (59.0%)	9092 (62.2%)		811 (58.9%)	806 (58.6%)	
ICSI	298 (21.5%)	4109 (28.1%)		298 (21.7%)	391 (28.4%)	
IVF+ICSI	269 (19.4%)	1405 (9.6%)		267 (19.4%)	179 (13.0%)	
Basal LH (mIU/mL, mean ± SD)	5.43 ± 3.93	5.48 ± 6.35	0.740	5.42 ± 3.92	4.62 ± 3.42	<0.001
Basal E2 (pg/mL, mean ± SD)	39.48 ± 51.04	62.52 ± 76.09	<0.001	39.51 ± 51.17	60.08 ± 62.69	<0.001
Basal FSH (U/L, mean ± SD)	5.08 ± 1.41	6.09 ± 3.55	<0.001	5.07 ± 1.41	6.02 ± 3.78	<0.001
Basal P (ng/mL, mean ± SD)	0.26 ± 0.31	0.31 ± 0.71	0.007	0.26 ± 0.31	0.29 ± 0.49	0.092
Total T (ng/mL, mean ± SD)	0.511 ± 0.29	0.26 ± 0.16	<0.001	0.04 ± 0.13	0.03 ± 0.12	0.048
Indicators on the day of embryo transferred						
E2 (pg/mL, mean ± SD)	260.56 ± 479.39	218.03 ± 343.61	<0.001	146.97 ± 330.72	170.38 ± 389.62	0.089
P (ng/mL, mean ± SD)	9.95 ± 11.95	9.74 ± 11.93	0.535	9.97 ± 11.96	10.19 ± 12.05	0.629

### Pregnancy Outcomes

Comparison of pregnancy outcomes between the two groups is shown in **Table 3**. The PCOS group displayed a shorter gestational age (P<0.001) and a slighter birth weight (P<0.001) than the non-PCOS group. For adverse pregnancy outcomes, the PCOS group had higher probabilities of GDM and PIH, (P<0.001), placenta and membrane abnormality (P<0.001), stillbirth (P<0.001), neonatal complications (P<0.001), and miscarriage (P<0.001) than the non-PCOS group. However, the PCOS group showed a lower probability of ectopic pregnancy(P<0.001) and birth defects (P<0.001) than the non-PCOS group.

**Table 3:** The pregnancy outcomes of live born singletons in women with and without PCOS after FET.

Outcomes	Before PSM			After PSM		
	PCOS group (N=1387)	Non-PCOS group (N=14606)	P	PCOS group (N=1376)	Non-PCOS group (N=1376)	P
Gender*			0.741			<0.001
Male	641 (46.2%)	6630 (45.4%)		635 (46.1%)	577 (41.9%)	
Female	586 (42.2%)	5940 (40.7%)		585 (42.5%)	731 (58.1%)	
Gestational age (wk)			<0.001			<0.001
<32	186 (13.4%)	2075 (14.2%)		182 (13.2%)	71 (5.2%)	
32–36	124 (8.9%)	769 (5.3%)		123 (8.9%)	39 (2.8%)	
≥37	1077 (77.6%)	11762 (80.5%)		1071 (77.8%)	1266 (92.0%)	
Birth weight			0.097			<0.001
<2500g	63 (4.5%)	504 (3.5%)		63 (4.6%)	29 (2.1%)	
2500-4000g	1092 (78.7%)	11261 (77.1%)		1085 (78.9%)	1164 (84.6%)	
>4000g	75 (5.4%)	865 (5.9%)		75 (5.5%)	121 (8.8%)	
Ectopic pregnancy	3 (0.2%)	63 (0.4%)	0.215	3 (0.2%)	6 (0.4%)	<0.001
GDM and PIH**	206 (14.8%)	1302 (8.9%)	<0.001	205 (14.9%)	96 (7.0%)	<0.001
Placenta and membrane abnormality^#^	24 (1.7%)	311 (2.1%)	0.265	24 (1.7%)	20 (1.5%)	<0.001
Birth defects	16 (1.2%)	223 (1.5%)	0.243	16 (1.2%)	27 (2.0%)	<0.001
Stillbirth	2 (0.1%)	5 (0.03%)	0.122	2 (0.1%)	0	<0.001
Complication of labor and delivery^$^	9 (0.6%)	94 (0.6%)	0.961	9 (0.6%)	9 (0.6%)	1.000
Neonatal complication	55 (4.0%)	373 (2.6%)	0.003	55 (4.0%)	41 (3.0%)	<0.001
Miscarriage	157 (11.3%)	1973 (13.5%)	0.022	153 (11.1%)	62 (4.5%)	<0.001

*Excluding excludes ectopic pregnancy and miscarriage cases; **GDM and PIH, Gestational Diabetes Mellitus and Pregnancy-Induced Hypertension; ^#^Including placenta previa and premature rupture of membrane. ^$^Including postpartum hemorrhage, amniotic fluid embolism, rupture of uterus and dysfunction of cord.

### Relationship Between Serum Sex Hormones and Adverse Pregnancy Outcomes

Logistic regression was performed to clarify the association between serum hormones and adverse pregnancy outcomes in patients with PCOS who underwent IVF. **Table 4** shows that after the adjustment for basal LH, basal E2, basal FSH, basal P, E2, and P levels on the day of embryo transfer, no factor was associated with GDM and PIH. The parity in PCOS group was associated with neonatal complications (including congenital anomalies, urogenital defects, jaundice, and pneumonia) (adjusted OR=1.202, 95% CI=1.002–1.443, P=0.048). The serum levels of basal P (adjusted OR: 1.211, 95% CI= 1.021–1.436, P=0.028) was associated with neonatal complications in women with PCOS (**Table 5**). Multivariate logistic regression revealed that parity (adjusted OR=1.201, 95% CI=1.057–1.366, P=0.005) and basal E2 (OR: 1.003, 95% CI=1.001–1.006, P<0.018) were related to an increased risk of miscarriage. Meanwhile, a high serum P level on the day of embryo transfer was associated with a significantly decreased risk of miscarriage in the PCOS group (adjusted OR=0.971, 95% CI=0.957–0.985, P<0.001) (**Table 6**).

**Table 4:** Risk factors of GDM and PIH in women with PCOS.

	Before matching				After matching			
	Crude OR	P	Adjusted OR	P	Crude OR	P	Adjusted OR	P
Maternal BMI (Kg/m^2^)	1.000 (1.000-1.000)	0.836	1.000 (1.000-1.000)	0.850	1.011 (1.001-1.020)	0.027	1.009 (0.999-1.020)	0.072
Parity (high order *vs.* first)	1.037 (0.995-1.081)	0.088	1.047 (1.003-1.092)	0.037	1.053 (0.937-1.184)	0.386	1.043 (0.925-1.176)	0.489
Cycle method (artificial *vs.* natural)	1.278 (1.125-1.453)	<0.001	1.104 (0.959-1.271)	0.168	0.835 (0.577-1.288)	0.338	0.726 (0.489-1.076)	0.111
Fertilization method (ICSI *vs.* IVF)	0.965 (0.858-1.085)	0.550	0.973 (0.863-1.096)	0.652	0.850 (0.640-1.130)	0.263	0.869 (0.651-1.159)	0.338
Fertilization method (IVF+ICSI *vs.* IVF)	1.097 (0.935-1.287)	0.258	1.118 (0.950-1.316)	0.180	0.928 (0.680-1.265)	0.635	0.956 (0.697-1.312)	0.071
Basal LH (mIU/mL)	0.982 (0.971-0.992)	0.001	0.999 (0.987-1.010)	0.842	0.985 (0.959-1.012)	0.278	0.995 (0.943-1.050)	0.855
Basal E2 (pg/mL)	0.997 (0.996-0.998)	<0.001	0.998 (0.997-0.999)	0.001	0.998 (0.995-1.001)	0.259	0.998 (0.994-1.002)	0.277
Basal FSH (U/L)	0.994 (0.978-1.010)	0.456	0.991 (0.974-1.009)	0.332	0.994 (0.946-1.046)	0.826	0.995 (0.943-1.050)	0.855
Basal P (ng/mL)	0.932 (0.818-1.042)	0.196	0.961 (0.865-1.067)	0.453	0.627 (0.284-1.383)	0.247	0.682 (0.307-1.518)	0.349
Total T (ng/mL)	1.000 (0.764-1.310)	0.998	1.007 (0.750-1.350)	0.965	0.820 (0.439-1.531)	0.532	0.860 (0.463-1.596)	0.632
Indicators on the day of embryo transfer								
E2 (pg/mL)	1.000 (1.000-1.000)	0.058	1.000 (1.000-1.000)	0.065	1.000 (1.000-1.000)	0.405	0.998 (0.994-1.002)	0.277
P (ng/mL)	1.006 (1.002-1.010)	0.003	1.006 (1.002-1.010)	0.006	1.008 (0.999-1.016)	0.096	1.008 (0.999-1.017)	0.079

**Table 5:** Risk factors of neonatal complications in women with PCOS.

	Before matching				After matching			
	Crude OR	P	Adjusted OR	P	Crude OR	P	Adjusted OR	P
Maternal BMI (Kg/m^2^)	1.000 (1.000-1.000)	0.943	1.000 (1.000-1.000)	0.943	1.000 (0.965-1.036)	0.996	0.999 (0.964-1.036)	0.966
Parity (high order *vs.* first)	1.036 (0.959-1.120)	0.372	1.054 (0.974-1.041)	0.193	1.194 (0.999-1.427)	0.051	1.202 (1.002-1.443)	0.048
Cycle method (artificial *vs.* natural)	1.466 (1.143-1.880)	0.003	1.379 (1.048-1.814)	0.022	1.612 (0.787-3.303)	0.192	1.585 (0.731-3.438)	0.244
Fertilization method (ICSI *vs.* IVF)	1.197 (0.969-1.478)	0.095	1.213 (0.979-1.504)	0.078	1.045 (0.646-1.689)	0.858	1.086 (0.665-1.774)	0.742
Fertilization method (IVF+ICSI *vs.* IVF)	1.045 (0.762-1.433)	0.786	1.085 (0.786-1.497)	0.620	1.090 (0.632-1.880)	0.757	1.192 (0.683-2.079)	0.537
Basal LH (mIU/mL)	0.989 (0.971-1.007)	0.243	0.997 (0.975-1.019)	0.789	0.990 (0.948-1.035)	0.660	1.001 (0.952-1.052)	0.967
Basal E2 (pg/mL)	0.998 (0.997-1.000)	0.060	0.999 (0.997-1.001)	0.604	1.000 (1.000-1.001)	0.060	0.999 (0.984-1.004)	0.722
Basal FSH (U/L)	1.012 (0.987-1.036)	0.352	1.011 (0.984-1.038)	0.431	1.011 (0.952-1.074)	0.714	1.010 (0.948-1.077)	0.749
Basal P (ng/mL)	1.016 (0.895-1.153)	0.807	1.027 (0.910-1.160)	0.664	1.174 (0.998-1.381)	0.053	1.211 (1.021-1.436)	0.028
Total T (ng/mL)	1.083 (0.718-1.634)	0.704	1.104 (0.716-1.701)	0.655	1.749 (0.730-4.188)	0.210	1.754 (0.737-4.171)	0.204
Indicators on the day of embryo transfer							
E2 (pg/mL)	1.000 (1.000-1.000)	0.454	1.000 (1.000-1.000)	0.471	1.000 (1.000-1.001)	0.060	1.000 (1.000-1.001)	0.080
P (ng/mL)	1.004 (0.996-1.012)	0.316	1.003 (0.996-1.011)	0.387	1.000 (0.983-1.016)	0.957	0.999 (0.983-1.016)	0.927

**Table 6:** Risk factors of miscarriage in women with PCOS.

	Before matching				After matching			
	Crude OR	P	Adjusted OR	P	Crude OR	P	Adjusted OR	P
Maternal BMI (Kg/m^2^)	1.000 (1.000-1.000)	0.793	1.000 (1.000-1.000)	0.835	1.006 (0.991-1.022)	0.418	1.005 (0.987-1.023)	0.590
Parity (high order *vs.* first)	1.169 (1.134-1.205)	<0.001	1.181 (1.145-1.218)	<0.001	1.176 (1.039-1.330)	0.010	1.201 (1.057-1.366)	0.005
Cycle method (artificial *vs.* natural)	0.999 (0.906-1.102)	0.985	1.031 (0.927-1.147)	0.569	0.855 (0.544-1.343)	0.496	0.918 (0.577-1.461)	0.718
Fertilization method (ICSI *vs.* IVF)	0.967 (0.877-1.066)	0.496	1.045 (0.946-1.154)	0.390	0.942 (0.675-1.313)	0.723	1.008 (0.720-1.411)	0.963
Fertilization method (IVF+ICSI *vs.* IVF)	0.972 (0.841-1.123)	0.699	1.100 (0.949-1.274)	0.207	1.056 (0.740-1.507)	0.765	1.131 (0.785-1.629)	0.509
Basal LH (mIU/mL)	0.998 (0.991-1.005)	0.633	0.995 (0.987-1.003)	0.238	0.978 (0.946-1.012)	0.209	0.970 (0.932-1.011)	0.148
Basal E2 (pg/mL)	1.000 (1.000-1.000)	0.294	1.000 (1.000-1.001)	0.155	1.001 (1.001-1.003)	0.052	1.002 (1.000-1.004)	0.019
Basal FSH (U/L)	1.011 (1.000-1.022)	0.052	1.010 (0.999-1.023)	0.087	0.948 (0.874-1.027)	0.190	0.970 (0.893-1.053)	0.467
Basal P (ng/mL)	1.036 (0.987-1.087)	0.149	1.035 (0.987-1.086)	0.155	0.815 (0.382-1.742)	0.598	0.829 (0.472-1.454)	0.513
Total T (ng/mL)	0.302 (0.209-0.436)	<0.001	0.346 (0.239-0.502)	<0.001	0.898 (0.446-1.809)	0.763	0.952 (0.476-1.904)	0.888
Indicators on the day of embryo transfer								
E2 (pg/mL)	0.999 (0.999-1.000)	<0.001	0.999 (0.999-1.000)	<0.001	0.999 (0.999-1.000)	0.076	1.000 (0.999-1.000)	0.085
P (ng/mL)	0.986 (0.982-0.990)	<0.001	0.986 (0.982-0.990)	<0.001	0.970 (0.956-0.984)	<0.001	0.971 (0.957-0.985)	<0.001

## Discussion

### Main Findings

To our knowledge, this is the first propensity score-matched study to identify the risk factors of adverse pregnancy outcomes in women with PCOS who received IVF treatment. PCOS is a common endocrine disorder that affects about 6%–10% of women and is characterized by hypotestosteronemia, hyperinsulinemia, high LH/FSH ratio, and obesity (18). One of its most prevalent consequences is oligo/amenorrhea anovulation, leading to infertility problems in women of childbearing age (19). In the past decades, IVF has increased the pregnancy rate in women with PCOS compared with that in non-PCOS controls. However, an increased risk of developing unfavorable pregnancy complications was reported in women with PCOS (20). In one study, PCOS was considered as an independent risk factor associated with late miscarriage in women treated with IVF (21). Although the negative effects of PCOS on the assisted reproductive outcome of IVF/ICSI in women with PCOS have been widely investigated, the association between serum sex hormone levels and pregnancy outcome remains unclear. Thus, the influence of each hormone on pregnancy outcomes in women with PCOS cannot be accurately elucidated. In the present work, we compared the serum sex hormone levels and pregnancy outcomes between women with and without PCOS after PSM. The results showed that the patients with PCOS had a higher maternal BMI, higher basal LH, and higher testosterone levels than the non-PCOS women. Our study also retrospectively analyzed the pregnancy outcomes of women with and without PCOS. Our primary finding is that patients with PCOS who received IVF had a short gestational age and an increased risk of GDM and PIH, neonatal complications, and miscarriage. Furthermore, parity and basal P were associated with neonatal complications. In women with PCOS who received IVF treatment, parity and high basal E2 level had a negative effect on miscarriage. Meanwhile, P level on the day of embryo transfer was a protective factor on miscarriage.

In our study, women with PCOS who underwent IVF had worse pregnancy outcomes compared with non-PCOS women. Among the patients with PCOS, those who underwent IVF had an increased risk of small gestational age and low birth weight compared with those who got pregnant spontaneously. Mostinckx et al. (22) compared the obstetric and neonatal outcome of *in vitro* maturation and controlled ovarian stimulation for assisted reproductive technology in patients with PCOS and found that women with PCOS who received *in vitro* maturation had a shorter gestational age than those who received controlled ovarian stimulation. By contrast, Liu and colleagues (20) reported that the gestational age was not significantly different between women with PCOS and without PCOS; however, the authors did not adjust for confounding factors when they analyzed the pregnancy outcomes of a cohort of 666 women with PCOS and 7012 controls using chi-square test. This phenomenon may explain the inconsistency between their results and ours. The newborn birthweight in the PCOS group was slightly lower than that in the non-PCOS group possibly due to the relatively short gestational age of women with PCOS. Our result was in line with the conclusion of previous studies. Sunkara et al. (23) found that women with PCOS had an increased risk of low birthweight; however, no specific risk factor was identified in their study. In another case-control research, Sir-Petermann and colleagues (24) observed that women with PCOS who had spontaneous pregnancy exhibited a higher prevalence of small gestational age and low birth weight compared with the control group. Han et al. (25) found that the incidence of low gestational age infants was higher in women with PCOS than in women with infertility due to tubal factors (25). A previous animal experiment showed that placenta insufficiency and prenatal exposure to sex steroids may be the reason for the small gestational age (26).

Obesity is one of the typical clinical characteristics of PCOS and may result from insulin resistance. Univariable logistic regression analysis found a positive correlation between maternal BMI and GDM and PIH in patients with PCOS who received IVF treatment. GDM and PIH are the two most common pregnancy complications. A high risk of GDM and PIH is frequently reported in women with PCOS (27, 28). GDM and PIH may result from some of the clinical characteristics of PCOS, such as polycystic ovaries, insulin resistance, and hyperandrogenism (29, 30). Early evidence suggested that obesity increases the risk of type 2 diabetes during pregnancy in patients with PCOS, and this finding was in agreement with our result (31). However, multivariable analysis indicated that maternal BMI was not associated with GDM and PIH. Kouhkan and colleagues (27) identified PCOS history as a risk factor for GDM in women with treated PCOS. Regardless of spontaneous pregnancy or IVF, patients with PCOS have a significantly increased risk of GDM during pregnancy due to the metabolic disorders caused by insulin resistance that lead to obesity and diabetes (32, 33). For PIH, women with PCOS undergoing IVF treatment have a higher risk of PIH than non-PCOS women. In the current study, logistic regression showed that maternal BMI was positively related to the risk of PIH in women with PCOS who underwent IVF. This finding was in agreement with Joham et al. (34), who analyzed the data from the Australian Longitudinal Study on Women’s Health and found that the incidence of hypertension was higher among obese women with PCOS than among lean women with PCOS. Palomba et al. (35) conducted a meta-analysis and found that women with PCOS had a three- to fourfold increased risk of PIH when BMI was not adjusted. According to the present multivariable logistic regression analysis, although obesity was not a risk factor of GDM and PIH, the patients with obesity must be given additional attention to prevent the occurrence of adverse pregnancy events.

In our study, we found that the risk of stillbirth was higher in the PCOS group than in the non-PCOS group. Our result is consistent with the findings of Valgeirsdottir et al. (36), who conducted a nationwide register-base cohort study and found that PCOS was associated with stillbirth. Two meta-analyses reported that PCOS is related to the risk of perinatal death, including stillbirth and early neonatal death (37, 38). However, the association between PCOS and stillbirth remains unclear.

The offspring of women with PCOS are at an increased risk of cardiovascular and other anomalies. Battaglia et al. (39) found that daughters born to patients with PCOS had an increased cardiovascular risk. Doherty and colleagues (40) demonstrated that the offspring of women with PCOS were at a high risk of postnatal hospitalizations, congenital anomalies, and urogenital defects. Our results suggested that the risk of neonatal complications in the offspring of women with PCOS is higher than that in the offspring of women with other infertile causes.

After PSM, the clinical miscarriage rate in women with PCOS was significantly different compared with that in non-PCOS women. However, we demonstrated that parity and serum basal E2 level were related to an increased risk of miscarriage, and a high serum P level on the day of embryo transfer was associated with a significantly decreased risk of miscarriage in the PCOS group. Progesterone is an essential hormone for maintaining early pregnancy and decidualized endometrium, relaxing uterine smooth muscle, improving uterine blood supply, and regulating immunity in early pregnancy (41). Supplement progesterone is widely used for miscarriage prevention and treatment of assisted reproductive technology. Miscarriage occurs in 15%–20% of all clinical pregnancies, and most of the causes remain unknown (42). In our article, we demonstrated that the serum progesterone level on the day of embryo transfer was a protective factor for miscarriage, and the estrogen level was related to an increased risk of miscarriage. Women with PCOS have a high risk rate of miscarriage; the increase in estradiol caused by endocrine disorders works synergistically with factors such as hyperinsulinemia, free insulin-like growth factors, and obesity, thus ultimately leading to infertility and miscarriage (43). Nevertheless, only a few studies focused on the prognosis of serum estrogen and progesterone levels on the day of embryo transfer for the pregnancy outcomes in patients with PCOS; large-scale studies are needed in the future. Compared with the non-PCOS group, the PCOS group had greater percentage of first-time pregnancy. However, parity was a risk factor of neonatal complication and miscarriage in our study. To date, limited studies have focused on parity and the occurrence of adverse pregnancy events in patients with PCOS. Additional research is needed to clarify this relationship in the future.

### Strengths and Weaknesses

The strengths of our study are as follows. First is the large cohort that focused on women with PCOS who underwent FET, which was adjusted the confounding factors by propensity score matching. Maternal age, paternal age, sperm origin, stage at cryopreservation, and number of embryos transferred were matched to reduce the potential bias when analyzing the risk factors between women with and without PCOS. Second, we conducted a comprehensive analysis to identify the risk factors of adverse pregnancy outcomes in women with PCOS. The sex hormone levels in women with PCOS greatly differ from those in women without PCOS. In this work, we analyzed the association of the sex hormones in patients with PCOS with GDM and PIH, neonatal complications, and miscarriage. We revealed the relationship between factors, such as maternal BMI, duration of infertility, parity, cycle method, fertilization method, basal serum of FSH, P, and E2, and adverse pregnancy events in patients with PCOS. However, this study also has several limitations. First, selection and information bias cannot be ruled out because of the retrospective design of this study. Second, this research is a single-center study, and the cases are all Chinese national. Hence, the relationship between the sex hormones and pregnancy outcomes must be validated in multiple centers. Third, PCOS patients are treated with drugs while undergoing IVF treatment, which can affect the serum level of sex hormones. Such drug treatment will lead to the development of patients with PCOS on the bright side, leading us to underestimate the impact of PCOS disease on IVF results. Fourth, owing to the observational nature of this study, the positive results are just a statistical correlation. Hence, causality cannot be established. Further investigation is needed to determine the underlying mechanism of the relationship between sex hormones and pregnancy outcomes in women with PCOS who received IVF treatment.

## Conclusion

This study evaluated the incidence of adverse pregnancy outcomes in women with and without PCOS. The results showed that women with PCOS have a higher risk of GDM and PIH, stillbirth, neonatal complications, and miscarriage than non-PCOS women. Neonatal complication was associated with parity and basal P level. Parity and basal estradiol were an increased risk factor of miscarriage, and serum progesterone level on the day of embryo transfer was a protective factor in women with PCOS.

## Data Availability Statement

The original contributions presented in the study are included in the article/supplementary material. Further inquiries can be directed to the corresponding author.

## Author Contributions

CY and YK conceived the study. ZN, SM, and SY extracted and analyzed the data. YL and WC contributed to the acquisition, analysis, and interpretation of the data. ZN and SM wrote the first draft of the manuscript which was revised by SY and LZ. This study was supervised by CY and YK. All authors contributed to the article and approved the submitted version.

## Funding

This work was supported by National Natural Science Foundation of China (82004408), Leading Project of Traditional Chinese Medicine of Shanghai Science and Technology Committee (19401930200) and Shanghai Sailing Program (20YF1448600).

## Conflict of Interest

The authors declare that the research was conducted in the absence of any commercial or financial relationships that could be construed as a potential conflict of interest.

## Publisher’s Note

All claims expressed in this article are solely those of the authors and do not necessarily represent those of their affiliated organizations, or those of the publisher, the editors and the reviewers. Any product that may be evaluated in this article, or claim that may be made by its manufacturer, is not guaranteed or endorsed by the publisher.
